# Unintentional injuries in children with disabilities: a systematic review and meta-analysis

**DOI:** 10.1186/s40621-015-0053-4

**Published:** 2015-09-15

**Authors:** Xiuquan Shi, Junxin Shi, Krista K. Wheeler, Lorann Stallones, Shanthi Ameratunga, Tom Shakespeare, Gary A. Smith, Huiyun Xiang

**Affiliations:** 1 Department of Epidemiology and Health Statistics, School of Public Health, Zunyi Medical College, Zunyi, Guizhou China; 2 Center for Injury Research and Policy, The Research Institute at Nationwide Children’s Hospital, The Ohio State University College of Medicine, 700 Children’s Drive, Columbus, OH 43205 USA; 3 Center for Pediatric Trauma Research, The Research Institute at Nationwide Children’s Hospital, The Ohio State University College of Medicine, Columbus, OH USA; 4 Colorado Injury Control Research Center, Colorado State University, Fort Collins, Colorado USA; 5 Section of Epidemiology and Biostatistics, School of Population Health, Faculty of Medical and Health Sciences, University of Auckland, Auckland, New Zealand; 6 Norwich Medical School, University of East Anglia, Norwich, UK; 7 Department of Pediatrics, College of Medicine, The Ohio State University, Columbus, OH USA

**Keywords:** Disability, Injury, Meta-analysis, Children

## Abstract

Children with disabilities are thought to have an increased risk of unintentional injuries, but quantitative syntheses of findings from previous studies have not been done. We conducted a systematic review and meta-analysis to assess whether pre-existing disability can increase the risk of unintentional injuries among children when they are compared to children without disability. We searched 13 electronic databases to identify original research published between 1 January 1990 and 28 February 2013. We included those studies that reported on unintentional injuries among children with pre-existing disabilities compared with children without disabilities. We conducted quality assessments and then calculated pooled odds ratios of injury using random-effects models. Fifteen eligible studies were included from 24,898 references initially identified, and there was a total sample of 83,286 children with disabilities drawn from the eligible studies. When compared with children without disabilities, the pooled OR of injury was 1.86 (95 % CI 1.65–2.10) in children with disabilities. The pooled ORs of injury were 1.28, 1.75, and 1.86 in the 0–4 years, 5–9 years, and ≥10 years of age subgroups, respectively. Compared with children without disabilities, the pooled OR was 1.75 (95 % CI 1.26–2.43) among those with International Classification of Functioning (ICF) limitations. When disability was defined as physical disabilities, the pooled OR was 2.39 (95 % CI 1.43–4.00), and among those with cognitive disabilities, the pooled OR was 1.77 (95 % CI 1.49–2.11). There was significant heterogeneity in the included studies. Compared with peers without disabilities, children with disabilities are at a significantly higher risk of injury. Teens with disabilities may be an important subgroup for future injury prevention efforts. More data are needed from low- and middle-income countries.

## Review

### Introduction

The 2013 State of the World’s Children report is focused on improving the lives of children with disabilities by promoting more inclusive societies where “physical, attitudinal, and political barriers are dismantled” (UNICEF 2013). The *Global Burden of Disease* estimate that 5.1 % of children worldwide (about 93 million) have moderate or severe disability is often cited (WHO 2011), but this estimate is not reliable given the substantial variation in disability definitions and surveillance/study methodologies (UNICEF 2013). From one country to another, the reported prevalence of disability in children ranges from 0.4 to 18.0 % (Maulik and Darmstadt 2007; Sinclair and Xiang 2008). It has been estimated that around 80 % of children with disabilities live in developing countries (WHO 2011). There is increasing recognition of disparities in health experienced by individuals with disabilities when compared with those without disabilities, including increased risks of violence (Jones et al. 2012) and unintentional injury (WHO et al. 2011). Injury is the leading cause of morbidity and mortality in children, therefore injury prevention rather than overprotection in this special population is important particularly as societies move toward greater inclusion (Wang et al. 2008; Kendrick et al. 2013b; UNICEF 2013).

The risk of violence against individuals with disabilities has been the subject of two meta-analyses (Hughes et al. 2012; Jones et al. 2012), including one among children with disabilities (Jones et al. 2012). Our previous meta-analysis in adults with disabilities demonstrated that they are at increased risk of unintentional injuries when compared with adults without disabilities (Shi et al. 2015). Children with disabilities are also thought to be at greater risk of unintentional injury (Xiang et al. 2014; Yung et al. 2014). Two prior reviews have included children, but neither has elucidated differences across age groups and disability subtypes nor did these studies include meta-analyses and summary measures of risk (Xiang et al. 2014; Yung et al. 2014). Age, because of its relationship to developmental ability and activity participation, is a very important factor when considering injury risk in children. It is not known if disability subtype plays a role in quantifying injury risk. There are substantial differences in the activities and injury patterns between adults and children, as well as considerable variation in the studies examining injury risk among children with disabilities.

We undertook a systematic review and meta-analysis to better quantify the risk and characteristics of injury among children younger than 18 years of age with disabilities. We synthesize the existing evidence to identify knowledge gaps and research priorities, so future injury prevention efforts can better serve children with disabilities.

### Methods

#### Search strategy

A search strategy was developed for 13 potential databases (Medline; Alt Health Watch, CINAHL, ERIC, PsycINFO, and Sport Discus via EBSCO; Scopus; CAB Abstracts, Global Health via CAB Direct; ISI Web of Knowledge; Cochrane Library; and Health Safety Science abstracts and Clinical Key) using the free text or keyword searches in any fields throughout the full texts. We used search terms from two categories related to disability (e.g., “disabilit*,” “limit*,” “disabl*,” “deficien*,” and “handicap*”); and injury (e.g., “injur*,” “hurt*,” “trauma,” “fall*,” and “wound*”). Additional strategies included hand searches of related journals, internet searches, and screening the reference lists of retrieved studies. Our search included all the related studies between 1 January 1990 and 28 February 2013 with English language abstracts. In this analysis, children were those younger than 18 years.

#### Definition of disability and injury

Disability was defined in a number of different ways in the included studies: (1) developmental disabilities; (2) emotional/mental/cognitive disabilities; (3) physical/sensory disabilities; (4) chronic disease with mention of functional limitations; (5) WHO International Classification of Functioning Disability and Health (ICF) impairments, limitations, and participation restrictions (Srinivasan et al. 2010; Brophy et al. 2008). A team discussion and decision process was utilized to create three subgroups of disability: physical disability, cognitive disability, and ICF-based definitions of disability.

Injury was defined as any injuries requiring medical attention in the 12 months preceding the interview. Not all studies included information about the types of injury, so we could not consider injury subtypes in our pooled analyses.

#### Literature selection

**Fig. 1:**
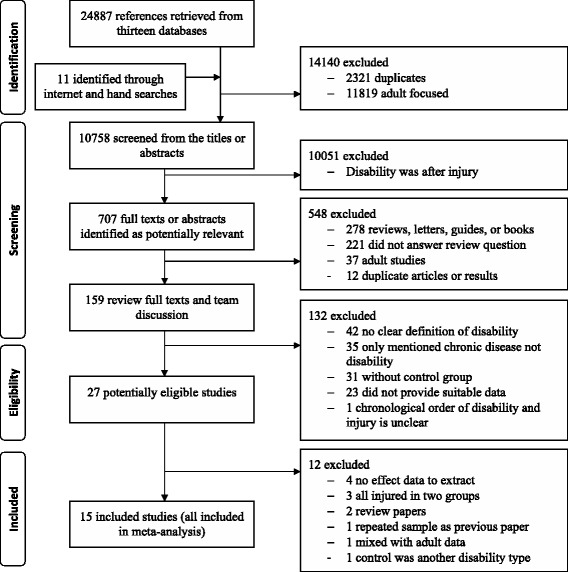
Flowchart of study selection

Details of our selected steps are shown in Fig. 1. All the retrieved studies were read and screened by two of five reviewers (XS, JS, KW, SL, and HX) in the first round of review. Any disagreements between the initial two reviewers were submitted to whole team for discussion and decision.

To be included in our meta-analysis, publications had to meet all the following inclusion criteria: (1) published as an original research article in a peer-reviewed journal with an English abstract; (2) investigated unintentional injuries among individuals with pre-existing disabilities; (3) reported age and a majority of participants were younger than 18 years old; (4) reported odds ratios (OR) or relative risks (RR) and confidence intervals (CI), or provided data so that these could be calculated for the disability variable; and (5) included clear definitions of disability and injury. The majority of the included studies did not specifically mention injury intent. When intentional injuries were mentioned, they were a subset of the total injuries and were excluded from the total injuries.

The following were reasons for exclusion: (1) published as review articles, in books, newspapers, magazines, or other commentaries and lacked original research data; (2) reported only fatal unintentional injuries or focused on intentional injuries (violence, crime, or suicide); (3) lacked a control group; (4) lacked demographic information or sufficient injury risk data; and (5) duplicate publications from the same study sample.

#### Quality assessment and data extraction

The quality of the included studies was assessed independently by two of the reviewers using the Strengthening the Reporting of Observational Studies in Epidemiology (STROBE) which has been used by others (Singh et al. 2011). The STROBE checklist contains 22 items which should be covered in epidemiology reports, with an emphasis on research methods (9 items) such as study design, data source, statistical methods, and bias. If an item was appropriately mentioned, a point was assigned (von Elm et al. 2007). After quality assessments, one reviewer (XS) extracted the following data: first author, publication year, study design, age, gender, research region, location at time of injury, the definitions and types of disabilities and injuries, number of disabilities and injuries (OR or RR and their CIs), and injury incidence using a standard data extraction form. A second researcher (JS) then checked data accuracy. When raw data from some studies were not reported, the corresponding authors were contacted to request data. Sufficient data were reported in the included studies for us to calculate the combined ORs by the different age subgroups and by different disability-type subgroups. Some included studies did not provide potential covariate risk factors, such as gender, family economic status, and other health conditions, which are related to disability and injury. These missing details meant that we were not able to estimate the adjusted risks and do a meta-regression analysis.

#### Data analyses

We first considered the characteristics of the included studies and then conducted heterogeneity tests to determine the appropriate approach for pooling the studies’ results. When heterogeneity (the degree of dissimilarity in the results of selected studies, *I*^*2*^ statistic) was statistically significant, we used random-effects models to compute the pooled ORs as opposed to fixed-effects models. We calculated pooled ORs and 95 % CIs and performed *Z* tests to evaluate the statistical significance of the pooled effects. We also produced pooled estimates for the different age groups and different types of disabilities (ICF-based disability, physical disability, and cognitive disabilities).

We conducted sensitivity analyses to evaluate the reliability of our results: showing the random-effects model and fixed-effects model results by dropping those studies with the highest and lowest ORs, and by dropping those studies with the largest and smallest sample sizes. Publication bias, i.e., studies with positive results are more likely to be published, was diagnosed by the funnel plot, Egger’s test, and Begg’s tests (Egger et al. 1997; Begg and Mazumdar 1994). We did all analyses using STATA software version 12.0 (StataCorp. TX, USA) with *p* values ≤0.05 considered significant (two-sided tests).

### Results

From 24,898 titles and abstracts, we identified 15 studies (Dunne et al. 1993; Leland et al. 1994; Sherrard et al. 2002; Xiang et al. 2005; Slayter et al. 2006; Chen et al. 2007; Mann et al. 2007; Lee et al. 2008; Sinclair and Xiang 2008; Raman et al. 2009; Ramirez et al. 2010; Brenner et al. 2013; Tsang et al. 2012; Zhu et al. 2012; Othman and Kendrick 2013) eligible for inclusion (Fig. 1). Ten studies used a cross-sectional design (Brenner et al. 2013; Chen et al. 2007; Lee et al. 2008; Mann et al. 2007; Raman et al. 2009; Sinclair and Xiang 2008; Slayter et al. 2006; Tsang et al. 2012; Xiang et al. 2005; Zhu et al. 2012), two studies used a case–control design (Dunne et al. 1993; Othman and Kendrick 2013), and three studies were cohort studies (Leland et al. 1994; Ramirez et al. 2010; Sherrard et al. 2002). Sample sizes ranged from 186 to 8,456,144 with a total sample size of 9,581,553, including 83,286 children with disabilities (not including three studies (Brenner et al. 2013; Sherrard et al. 2002; Ramirez et al. 2010) in which the authors did not provide the exact number of individuals with disability). Across the 15 studies, 2,032,685 children were injured (including 22,306 injured children with disabilities).

All studies had mixed gender samples. A gender difference in injury risk existed and the pooled injury risk for males with disability was a little higher than that for females with disability (OR = 1.20, 95 % CI 1.06–1.36). Most studies used a broad age range, generally from 0 to 18 years, however three studies focused on young children (Leland et al. 1994; Lee et al. 2008; Othman and Kendrick 2013) and five studies focused on school-age children (Sherrard et al. 2002; Raman et al. 2009; Xiang et al. 2005; Ramirez et al. 2010; Tsang et al. 2012) (Table 1). We also included two studies (Slayter et al. 2006; Ramirez et al. 2010) which had small percentages of young adults ages 19 and 20, because these young adults could not be separated in the analysis from those under 18 years. Geographically, the WHO region of the Americas was heavily represented, with ten studies in USA and one in Canada (Raman et al. 2009), and the remaining four studies were from the WHO Asia and Western Pacific region (China, Hong Kong, Iraq, and Australia) (Othman and Kendrick 2013; Sherrard et al. 2002; Tsang et al. 2012; Zhu et al. 2012). No eligible studies were found in the WHO Europe and Africa regions.

**Table 1:** Characteristics of included papers

First author/year	Design	Data source	Age	Definition and type of disability	Pre-existing disability determination	Definition and type of injury	% injured with disabilities/without disabilities^a^	Quality assessment^b^
Dunne RG, 1993	CC	National Health Interview Survey, 1988	0–17 years	Developmental disability, mainly cognitive disability	Developmental delays not likely the result of injury	Injury requiring medical attention in past 12 months reported by care giver	28.7/26.2 %	17–19
Leland NL, 1994	CO	Preschool children in two day care programs	30–72 months	Medical diagnosis of physical or cognitive disability; 63 % with cognitive disability	Enrolled in one of two day care programs based on disability	Day care injury logs as required by state law	4.8/2.5 %	20–21
Sherrard J, 2002	CO	Australian Child and Adolescent Development program, 1990–1991 and 1995–1996	4–18 years	Cognitive disability, intelligence quotient <70	Biopsychosocial data collected in 1990–1991, injury assessed in 1995–1996	Medically attended injuries in past 12 months reported by care giver	—	19–20
Xiang H, 2005	CS	National Health Interview Survey, 2000–2002	5–17 years	ICF—limitations in social activities because of chronic physical or mental conditions	Disabling condition for at least 1 year before the interview	Medically attended injuries in past 3 months	4.1/2.5 %	21–21
Slayter EM, 2006	CS	Medicaid-eligible children in 26 states, 1999 eligibility and claims data	1–20 years	Cognitive disability, ICD-9-CM codes 317–319	Cognitive disability codes unrelated to injury	ICD-9-CM injury codes in Medicaid claims data	36.9/23.5 %	20–22
Chen G, 2007	CS	Ohio Medicaid claims data 2002	0–12 years	ICF—limitations in social activities because of chronic physical or mental conditions	Medicaid designated disability, limitations as the result of a chronic condition	ICD-9-CM codes for burns 940–949	1.03/0.77 %	20–22
Mann JR, 2007	CS	South Carolina Medicaid claims data, 2002–2003	1–18 years	Hearing loss, ICD-9-CM codes 389.0–389.9	Hearing loss diagnosis in both 2002 and 2003, injury in 2003	ICD-9-CM codes for injuries, Barell Matrix categories	17.7/8.6 %	21–21
Lee LC, 2008	CS	National Survey of Children’s Health, 2003–2004	3–5 years	Learning disability	Disabilities unrelated to injury	Medically attended injuries in the past year	16.6/12.2 %	20–22
Sinclair SA, 2008	CS	National Health Interview Survey, 1997–2005	0–17 years	ICF—limitations in activities, excluding those with multiple disabilities	Excluded children who had an injury less than 1 year before the interview that resulted in a disability	Medically attended injuries in past 3 months	3.8/2.5 %	21–21
Raman SR, 2009	CS	Health behavior in school-age children survey, Canada 2002	Grades 6–10	ICF—long-term disability with participation and activity limitations	Disability is reported to be long term, past year injuries with reported consequences	Student self-report of medically attended injuries in the past 12 months	67.4/51.4 %	20–20
Ramirez M, 2010	CO	35 schools in urban district of Los Angeles, 1994–1998, *n* = 147,460	5–19 years	Qualified for special education services by California Department of Education	Students enrolled for services, subsequent school injuries	Injuries during school activities	3.8/1.5 %	19–21
Brenner RA, 2013	CS	National Electronic Injury Surveillance System, 2006–2007	0–17 years	Autism, blindness, cerebral palsy, deafness or trouble hearing, intellectual disability, ADD, ADHD, learning disability	Caregivers surveyed, disabilities unrelated to injury	All non-work unintentional injuries	10.4/10.5 %	19–20
Tsang SL, 2012	CS	Students in 2 mainstream and 3 special schools	6–12 years	Cognitive disability, intelligence quotient <70	Caregivers surveyed, disability unrelated to injury	Unintentional household injuries; home	61.6/32.0 %	19–19
Zhu HP, 2012	CS	Registry database of China Disabled Persons’ Federation	1–14 years	ICF	Causes of limitations were known	All medical attention injuries in the past year; home, school, other locations	10.2/4.4 %	20–20
Othman N, 2013	CC	Burn center and admitted patients in a children’s hospital in Iraq	0–5 years	Visual or hearing impairment, epileptic seizures, learning disabilities, walking problems	Reason for admission is known, excluded those with previous burn injury	Burns; home	—	17–18

*CC* case–control study, *CO* cohort study, *CS* cross-sectional study, *ICF* International Classification of Functioning, Disability and Health, *ICD-9-CM* International Classification of Disease, Ninth Revision, Clinical Modification, *ADD* attention deficit disorder, *ADHD* attention deficit hyperactivity disorder

^a^Some references did not provide the proportion injured or lacked data to calculate percentages

^b^Number of items among the 22 items in STROBE checklists judged by two reviewers

Not all of the 15 studies included comparisons regarding the location, activity, or cause of injury. Older studies were less likely to include this information, two papers reported injuries that occurred only at home (Othman and Kendrick 2013; Tsang et al. 2012), two reported only daycare and school injuries (Leland et al. 1994; Ramirez et al. 2010), four included a mix of injury locations (Brenner et al. 2013; Raman et al. 2009; Sinclair and Xiang 2008; Zhu et al. 2012), and seven papers did not clearly mention the location at time of injury (Dunne et al. 1993; Sherrard et al. 2002; Xiang et al. 2005; Slayter et al. 2006; Chen et al. 2007; Mann et al. 2007; Lee et al. 2008). When reported, home was the most common injury location followed by school. Two studies reported similarities in the injury patterns in children with and without disabilities (Brenner et al. 2013; Sinclair and Xiang 2008). Two studies reported higher proportions of burn injury (Sinclair and Xiang 2008), and two studies were focused on burn injuries only (Chen et al. 2007; Othman and Kendrick 2013). Others reported fewer sports-related injuries among children with disabilities compared to those without disabilities (Raman et al. 2009; Zhu et al. 2012; Sinclair and Xiang 2008). Intentional injuries were specifically excluded in three studies (Brenner et al. 2013; Tsang et al. 2012; Zhu et al. 2012), and intent was not mentioned in six of the studies (Chen et al. 2007; Dunne et al. 1993; Leland et al. 1994; Othman and Kendrick 2013; Sinclair and Xiang 2008; Xiang et al. 2005). For those reporting on school injuries (Raman et al. 2009; Ramirez et al. 2010), nearly one third of the injuries were the result of assaults/fights, and one of these studies reported a higher rate of assault/fight-related school injury among children with disabilities (Raman et al. 2009). One of the included studies references a companion paper which describes injury intent; intentional injuries were 15 % of the total injuries, and the rate of intentional injury was higher among those with disabilities (Sherrard et al. 2001). The intentional injuries in these papers were excluded from our pooled estimates.

Thirteen studies reported injury rates in children with disabilities (some papers did not report the exact rates but provided the data so that the rates could be calculated). Injury rates for the children with disabilities ranged from 1.0 to 67.4 % (median 10.4 %), and in the control group ranged from 0.8 to 51.4 % (median 8.6 %). We found large variation and heterogeneity in both groups, so we did not calculate pooled injury percentages. Instead, we calculated pooled ORs of injury to estimate risk.

The characteristics of included studies are listed in Table 1. The quality of studies was assessed using the STROBE checklist, and all the included studies met at least 17 items and most had 19–21 items in the checklist. Items most frequently missing were determination of bias, selection of participants, explanation of the quantitative variables, and how the study sample size was determined.

**Fig. 2:**
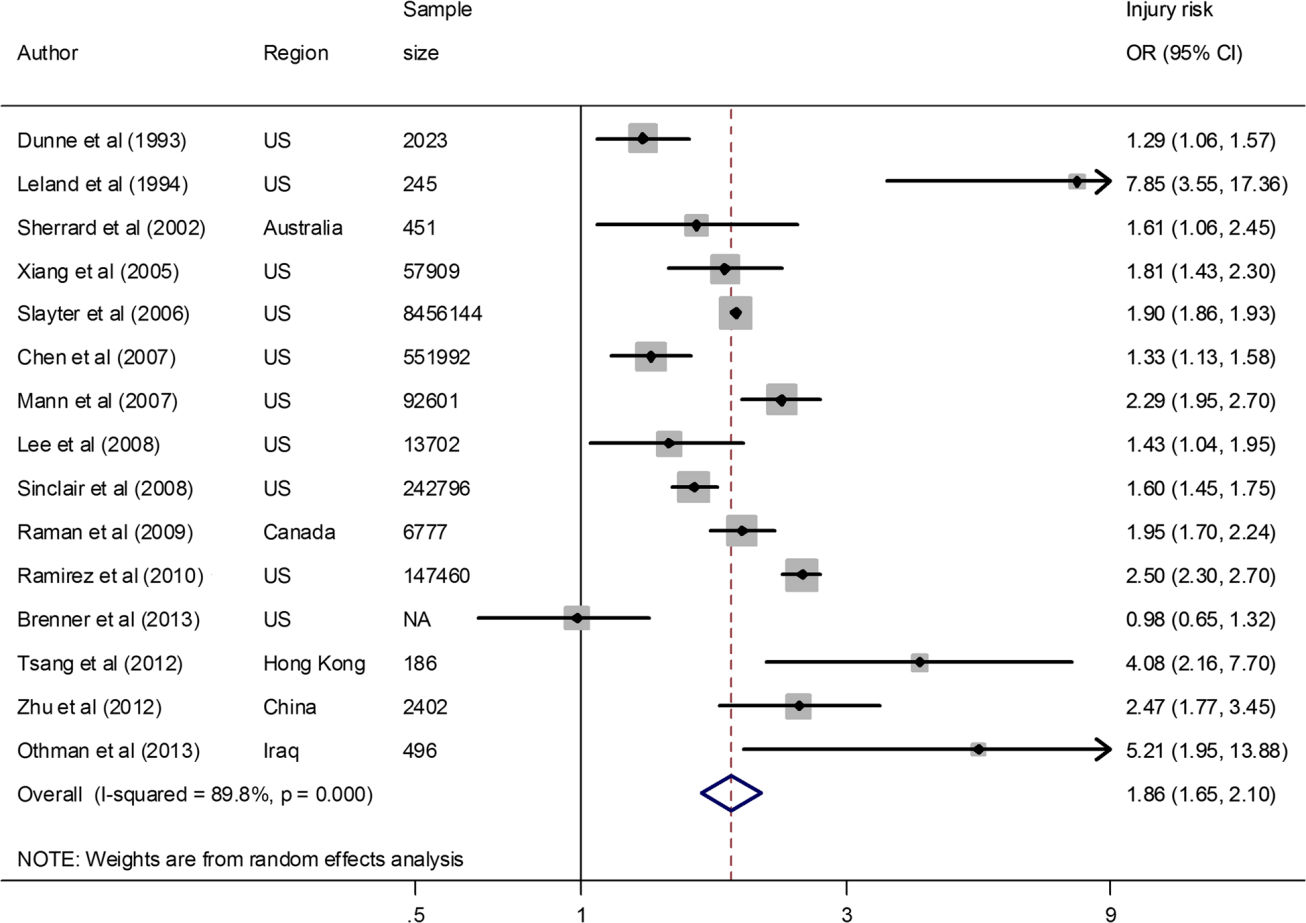
Overall analysis of injury risk against children with disabilities. *NA* not available, *OR* odds ratio, *CI* confidence interval. The ORs and CIs were computed using original numbers of disabilities and injuries. If the original data could not be extracted, we used the crude ORs and CIs rather than adjusted ORs and CIs

In our meta-analysis, we used random-effects models to estimate the pooled ORs because all of the *p* values of *Q* tests were <0.001 and the *I*^*2*^ results were all greater than 70 %, indicating that heterogeneity should be considered statistically significant. Of the 15 included papers, the *I*^*2*^ was 89.8 %, the combined OR was 1.86 (95 % CI 1.65–2.10), and the *Z* value was 10.07, *p* < 0.001 when testing whether the pooled effect was equal to 1 (Table 2 and Fig. 2).

**Table 2:** Results of the sensitivity analyses

Analyzed databases	Detailed databases^a^	Included studies	OR and CI	Overall *Z* test and *p* value	*I* ^2^ and CI (%)
Overall	All eligible papers	15	1.86(1.65–2.10)	*Z* = 10.07, *p* < 0.001	89.8(84.8–93.1)
	All eligible papers (fixed-effects model)	15	1.90(1.87–1.94)	*Z* = 73.67, *p* < 0.001	89.8(84.8–93.1)
Studies with the most variation in effect were dropped	Excluded max-effect paper	14	1.81(1.61–2.03)	*Z* = 9.87, *p* < 0.001	89.6(84.3–93.1)
	Excluded min-effect paper	14	1.92(1.71–2.17)	*Z* = 10.70, *p* < 0.001	89.5(84.1–93.0)
	Excluded max- and min-effect papers	13	1.87(1.67–2.10)	*Z* = 10.55, *p* < 0.001	89.2(83.4–93.0)
Studies with the most variation in sample size were dropped	Excluded max-sample size paper	14	1.91(1.60–2.28)	*Z* = 7.22, *p* < 0.001	90.5(85.8–93.6)
	Excluded min-sample size paper	14	1.82(1.61–2.05)	*Z* = 9.70, *p* < 0.001	90.1(85.2–93.4)
	Excluded max- and min-sample size papers	13	1.85(1.55–2.20)	*Z* = 6.76, *p* < 0.001	90.9(86.2–93.9)

*OR* odds ratio, *CI* confidence interval, *max* maximum, *min* minimum

^a^Random-effects models were used to combine the effects unless otherwise specified

**Fig. 3:**
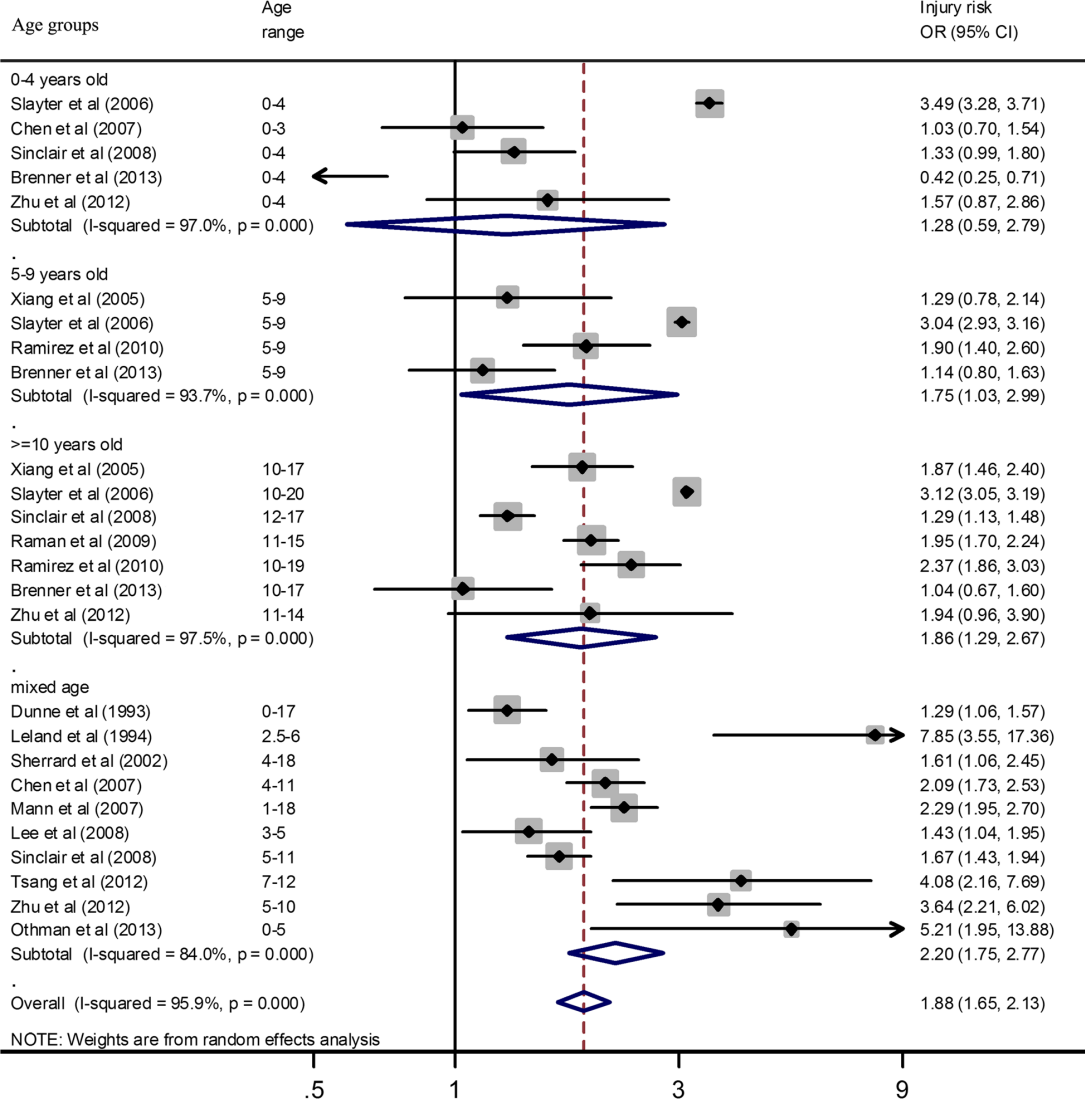
Injury risk estimates in children with disabilities according to age group. *OR* odds ratio, *CI* confidence interval. The ORs and CIs were computed using original numbers of disabilities and injuries. If the original data could not be extracted, we used the crude ORs and CIs rather than adjusted ORs and CIs

Results of the subgroup analyses by age group are shown in Fig. 3. For the four age subgroup analyses, all pooled results are from random-effects models. The overall *I*^*2*^ was 95.9 % (95 % CI 94.8–96.7 %) and the OR was 1.88 (95 % CI 1.65–2.13) which was statistically significant, *Z* = 9.67, *p* < 0.001. The *I*^*2*^, the measure of heterogeneity, ranged from 84.0 to 97.5 %. The OR of injuries increased with increasing age. For the 0–4-year-old group, the pooled OR was not significant [1.28 (95 % CI 0.59–2.79), *Z* = 0.63, *p* = 0.531]. For the 5–9-year-old group, the pooled OR reached statistical significance [1.75 (95 % CI 1.03–2.99), *Z* = 2.07, *p* = 0.039]. For the ≥10-year-old subgroup, the pooled OR was significant [1.86 (95 % CI 1.29–2.67), *Z* = 3.35, *p* = 0.001]; similarly, in the mixed-age subgroup (0–18 years old), the combined OR was significant [2.20 (95 % CI 1.75–2.77), *Z* = 6.68, *p* < 0.001]. The mixed-age group included data from studies where there were not enough data to separate the subjects into one of the three age subgroups.

**Fig. 4:**
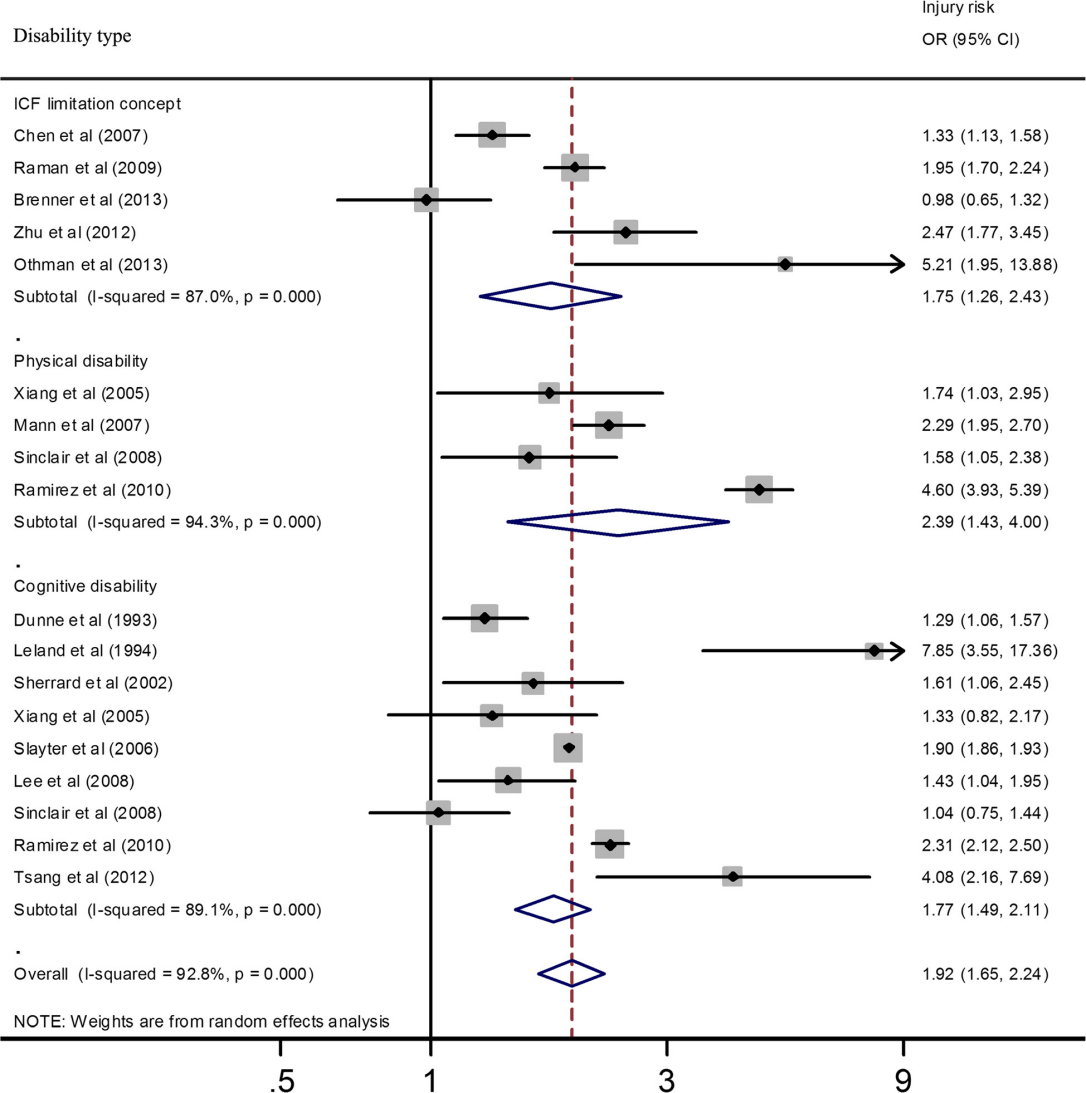
Injury risk in children with disabilities according to disability type. *OR* odds ratio, *CI* confidence interval. The ORs and CIs were computed using original numbers of disabilities and injuries. If the original data could not be extracted, we used the crude ORs and CIs rather than adjusted ORs and CIs

Subgroup pooled results for different types of disabilities are shown in Fig. 4. For the five studies that used ICF limitation questions, the *I*^*2*^ was 87.0 % (95 % CI 72.0–94.0 %) and the pooled OR was 1.75 (95 % CI 1.26–2.43) [*Z* = 3.34, *p* = 0.001]. For the four studies reporting physical disabilities, the *I*^*2*^ was 94.3 % (95 % CI 88.4–97.2 %) and the combined OR was 2.39 (95 % CI 1.43–4.00) [*Z* = 3.33, *p* = 0.001]. For the nine studies reporting cognitive disabilities, the *I*^*2*^ was 89.1 % (95 % CI 81.5–93.6 %) and the combined OR was 1.77 (95 % CI 1.49–2.11); *Z* = 6.40; *p* < 0.001. Three studies reported both physical disabilities and cognitive disabilities, and those data were included in a separate subgroup analysis (Xiang et al. 2005; Sinclair and Xiang 2008; Ramirez et al. 2010). The overall *I*^*2*^ was 92.8 % (95 % CI 90.1–94.8 %) and the OR was 1.92 (95 % CI 1.65–2.24) [*Z* = 8.45, *p* < 0.001]. (Note: The small difference in the overall effects shown in Figs. 3 and 4 was due to different ways the data were grouped. Not all studies were included in both the age and disability subgroup analyses).

To assess the reliability of our results, we conducted sensitivity analyses (Table 2), comparing the pooled estimates produced using two types of models (a random-effects model versus a fixed-effects model). We also calculated the pooled estimates after we excluded those studies with the most variation in effect or sample size. We found that the overall ORs changed little, so our meta-analysis results appeared reliable. We also tested for publication bias among our sample of studies using two approaches. The first approach was the funnel plot; asymmetry in the funnel plot indicates bias (Harbord et al. 2006). We found that there was good symmetry, though some points were out of the area of the CI (figure not shown). The second approach included the Egger’s and Begg’s tests, which conclude that bias exists if the intercept for the regression is different from zero at the 0.05 level (Begg and Mazumdar 1994; Harbord et al. 2006). The result of Begg’s test was *Z* = 0.89, *p* = 0.373 in all included studies; while the result of Egger’s test was *t* = 0.01, *p* = 0.990. Both plots and tests indicated that publication bias in our meta-analysis was not a substantial issue.

### Discussion

Findings from our meta-analysis of 15 studies indicate that children with disabilities are at increased risk of unintentional injuries. The pooled OR of unintentional injuries was higher in individuals with physical disabilities (OR = 2.39) compared with those with cognitive disabilities (OR = 1.77). We also found increased odds of injury with increasing age. Results of this meta-analysis, along with the meta-analysis of violence against children with disabilities, provide evidence that injury prevention among children with disabilities, both unintentional and intentional injury, merits attention in the injury prevention field (Jones et al. 2012). Recent research has documented that in the USA alone, the percentage of children with disabilities rose 16 % between 2001 and 2011 (Houtrow et al. 2014).

Age is well known as an important modifier in injury-related research. Children are not just small adults; their physical and cognitive abilities, degrees of dependence, need for supervision, activities and risk behaviors all change rapidly with their growth. As children develop, their curiosity and wish to explore the world increase significantly while they only have limited capacities to understand or respond to danger (Bartlett 2002). Children’s development and behaviors are therefore highly associated with injury risk. Injury characteristics differ at different ages. Poisoning, for example, is linked to the grasping and mouth-exploratory behaviors of children ages 1–3 years; falls are particularly related to the stage of learning to walk, while burns from hot liquids have previously been found to be higher among children ages 12–18 months (Agran et al. 2003). Our study supports this association between age and injury risk in children with disabilities. We found that the pooled ORs of injury were 1.28, 1.75, and 1.86 in 0–4, 5–9, and ≥10-year-old age subgroups, respectively. Some researchers have reported that among children with disabilities, occurrence of injuries decreased with increasing age (Limbos et al. 2004; Ramirez et al. 2004), but these studies were focused on injuries in the school environment. However, our results are consistent with the study of Petridou and colleagues, who found that the injury OR for children with disabilities increased with increasing age (Petridou et al. 2003). A similar result was also reported in Chen and colleagues’ study of burns risk in children with disabilities younger than 12 years old (Chen et al. 2007).

Similar to our finding in adults with disabilities, children with physical disabilities had the greatest odds of injury (OR = 2.39). Children with ICF limitations and cognitive disabilities had similar ORs (1.75 and 1.77, respectively). Our meta-analysis among adults with disabilities produced inconclusive evidence about injury risk in adults with cognitive limitations (Shi et al. 2015). Ramirez and colleagues’ cohort study of 269,000 school children reported that children with physical disabilities (orthopedic and sensory) were more likely to suffer injuries than those with cognitive disabilities (Ramirez et al. 2010).

In addition to injury risk differences across age groups and disability types, our meta-analysis showed that gender was also a significant modifier. The pooled OR of injury in boys with disabilities was 1.21 times greater than that in girls with disabilities. Reasons for this gender difference may include the fact that boys usually have higher activity levels, more risk behaviors, and are less restrained by parents (WHO, UNICEF 2008).

Our meta-analysis has several limitations. First, other socioeconomic status factors, such as family income and race, also potentially affect the injury risk in children with disabilities; however, we could not examine the pooled effects because only a limited number of original studies included those injury risk factors. A second limitation is the substantial methodological and statistical heterogeneity seen in the selected studies. Much of the methodological heterogeneity was due to the varied definitions of disability among these studies. In the pooled analysis, we estimated injury risk by physical disability, cognitive disability, and ICF disability subgroups. Even within these subgroups, disability definitions varied among different studies. Physical disability included sensory impairments, limb disabilities, and work limitations, while cognitive disability included intellectual disabilities, mental health disabilities, and learning disabilities. Future research in this area should use a consistent definition of disability so results from multiple studies can more readily be compared and pooled. More recent studies have used the ICF disability definition, which includes not only impairments but also activity limitations and participation restrictions (Wasiak et al. 2011). Although ICF concepts and disability definition are still evolving; they can provide a standardized terminology for epidemiological studies to achieve comparability of data. Some other sources of heterogeneity in our meta-analysis were significant variations in sample sizes, research study periods, and the age range of enrolled children. A third limitation is that the included studies came mostly from high-income countries and regions (USA, Canada, Australia, and Hong Kong), while low-income and middle-income countries have 80 % of the world’s disabled population, generally higher rates of injury, and fewer health services than developed countries (WHO et al. 2011). Research about injuries in children with disabilities in low- and middle-income countries is scarce. Although a few included studies were from middle-income countries (China and Iraq), the potential value of additional rigorous data on injury risk in children with disabilities from developing countries should not be ignored. A fourth limitation is related to grey literature (unpublished academic studies such as theses and dissertations). While we attempted to include such studies through internet searches, no such studies were found. Finally, it is not clear from the included studies if there are strong differences between children with disabilities and children without disabilities in the patterns of injury (location, activity, and mechanism of injury). Occupational injuries were considered in our adult study, but we did not find any studies which considered work-related injuries among adolescents.

Despite the heterogeneity of the included studies, several of our implemented research steps should have strengthened the reliability of our results. First, we had strict inclusion and exclusion criteria. Second, our literature selection procedures followed the recommended standard steps of systematic review and meta-analysis (Manchikanti et al. 2009; Moher et al. 2009). We reached selection decisions through two independent rounds of review and one round of team discussion when needed. Third, the two reviewers’ appraisals of original studies were based on the STROBE checklist, which showed that the quality of included studies was moderate to high (17–22 items were mentioned from a total 22 items).

By estimating the pooled injury risk of injuries in children with disabilities, this study highlights the need for interventions. A number of recent systematic reviews (Kendrick et al. 2013a; Kendrick et al. 2013b; Pearson et al. 2012) and reports (WHO, UNICEF 2008; UNICEF 2009) have described child injury prevention strategies. The 2008 World Report on Child Injury Prevention has chapters based on different external causes of injury and possible interventions for use in both high and low resource settings (WHO, UNICEF 2008). Similarly, Kendrick et al. reported on effective parenting interventions (Kendrick et al. 2013a) and home safety interventions (Kendrick et al. 2013b) in two recent systematic reviews. A 2012 systematic review of educational programs aimed at preventing unintentional injury during outdoor play reported mixed results (Pearson et al. 2012). While children with higher risks of injury were considered in these systematic reviews of interventions, children with disabilities were not one of the higher risk groups considered. Sherrard et al. reported on a small number of studies which evaluated injury prevention among both adults and children with cognitive disabilities (Sherrard et al. 2004). Despite the recognition in the disability community of the need for interventional studies (WHO et al. 2011; Xiang et al. 2014; Yung et al. 2014), by and large, rigorous evaluation studies of injury prevention interventions in children with disabilities could not be found in the literature. Some researchers have recommended using national injury surveillance systems to identify those risk factors which might be specific to children with disabilities (Gaebler-Spira and Thornton 2002).

Currently, it is not clear if different prevention strategies are needed for children with disabilities. Specific interventions for children with disabilities might potentially include actions to provide assistive devices and modify inaccessible or hazardous environments (passive interventions). In the former category would be appropriate wheelchairs and other mobility devices, as well as tools to enhance ability to reach. In the latter category would be provision of curb cuts and safe road crossings and barrier removal in the home. UNICEF’s 2013 State of the World’s Children report focused on enhancing societal participation of children with disabilities and calls for universal design in “all children’s environments—early childhood centers, schools, health facilities, public transport, playgrounds, and so on” (UNICEF 2013). Environmental factors, including stairs, curbs, and rough terrain, were identified as injury trigger factors in a study of pediatric mobility aid-related injuries (Barnard et al. 2010). Reducing the use of institutionalization, increasing support for families and involving children with disabilities indecision making are also addressed in the UNICEF report (UNICEF 2013). Future studies considering intervention effectiveness should include children with disabilities. As well, future research should be geared toward enhancing social participation and improving the safety of children’s environments, as opposed to restricting activities. The goal is to improve the quality of life for children with disabilities, and at stake is the principal of equity (UNICEF 2013).

## Conclusions

Our systematic review and meta-analysis showed that children with disabilities had a greater risk of unintentional injuries, especially children with physical disabilities. Teens were at a greater risk than younger children with disabilities. Future research on injuries in children with disabilities should focus on passive interventions (Simpson and Nicholls 2012) to prevent both intentional and unintentional injuries. More high-quality intervention and evaluation research is needed so that parents, teachers, healthcare, and other social service providers can choose evidence-based interventions to reduce injury risk in children with disabilities.

## Abbreviations

ICF: International Classification of Functioning
STROBE: Strengthening the Reporting of Observational Studies in Epidemiology
WHO: World Health Organization
